# Supplementary material for: miR-22 Modulates Lenalidomide Activity by Counteracting MYC Addiction in Multiple Myeloma
Source: Cancers (Basel). 2021 Aug 29;13(17):4365. doi: 10.3390/cancers13174365 (PMC8431372; doi:10.3390/cancers13174365)
Supplement: Supplementary file 1 [file cancers-13-04365-s001.zip › cancers-1349609-supplementary.pdf]

SUPPLEMENTARY FIGURE S1

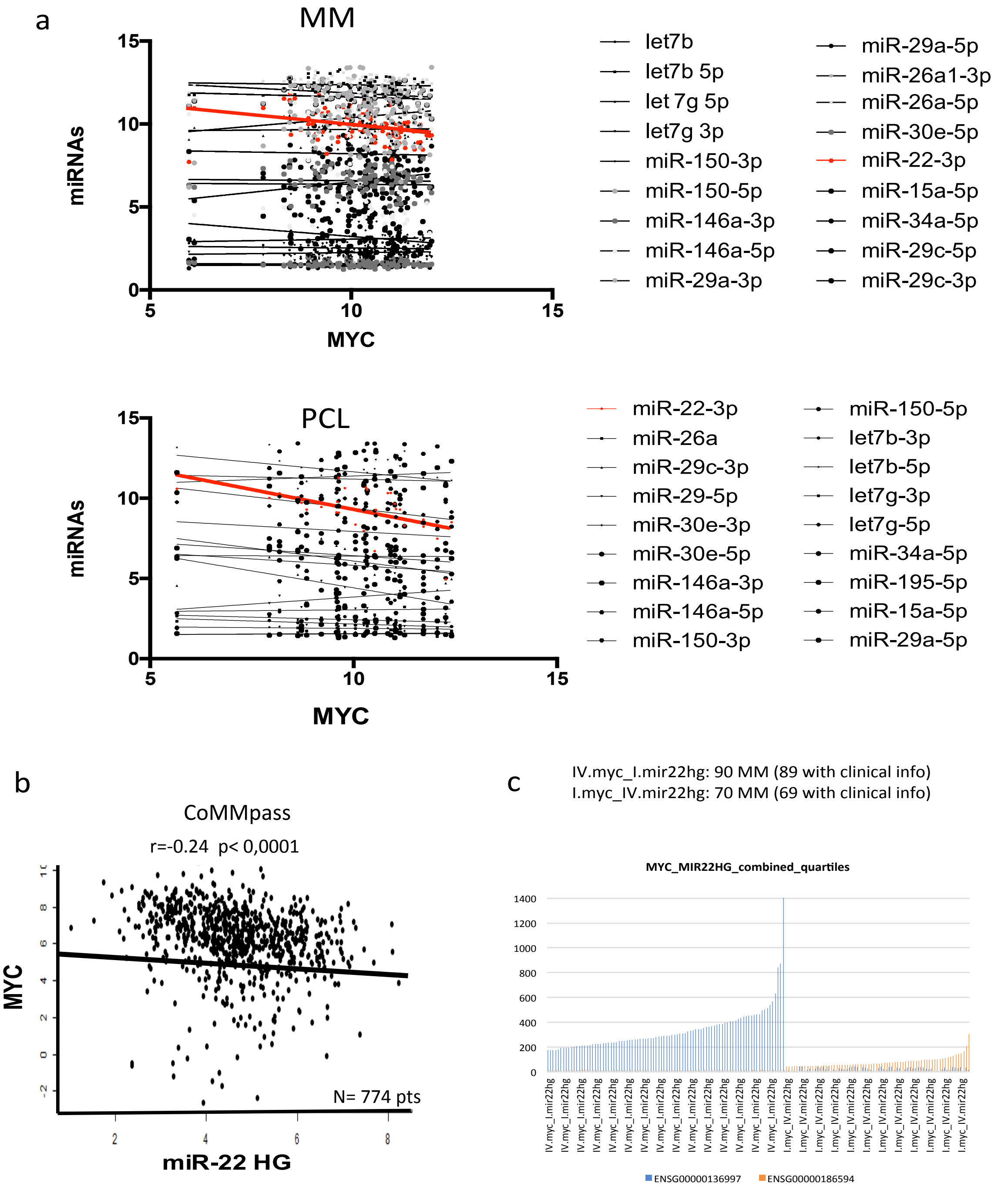


**Supplementary Figure S1: In silico prediction of MYC repressed miRNAs in MM.** a) Graphs of correlations between endogenous mRNA expression levels of MYC and predicted MYC-repressed miRNAs, in patient's MM and PCL cases from proprietary dataset (GSE73454 and GSE70254). b) 774 MM samples of the MMRF-CoMMpass dataset were stratified accordingly to MYC and MIR22HG expression levels, inversely combining the two extreme quartiles of each group.

SUPPLEMENTARY FIGURE S2

a

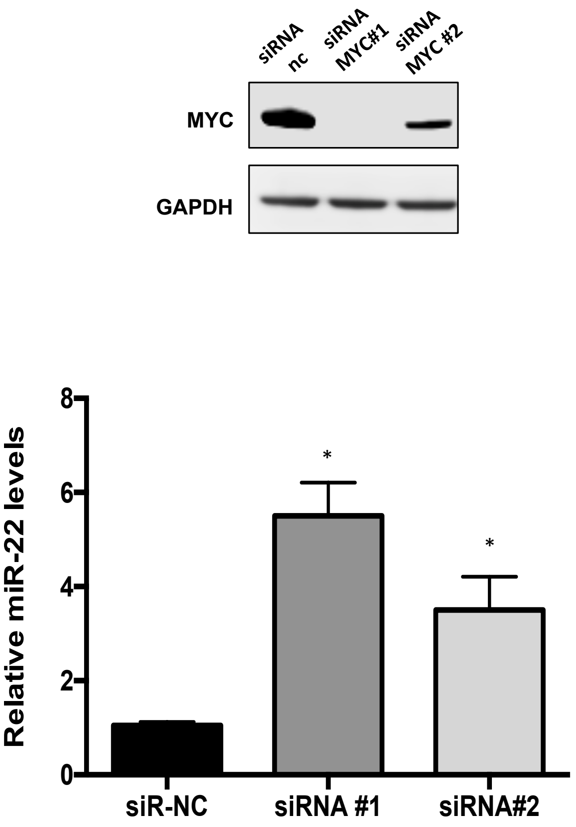


**Supplementary Figure S2: MYC negatively affects miR-22 expression in MM:** a) AMO1 cells were transfected with two different MYC siRNAs or with negative control. Immunoblot of MYC was performed 48h after transfection. GAPDH was used as a loading control. Results are average ±SD of three independent experiments performed in triplicate. *P<0.05

SUPPLEMENTARY FIGURE S3

a 3'UTR

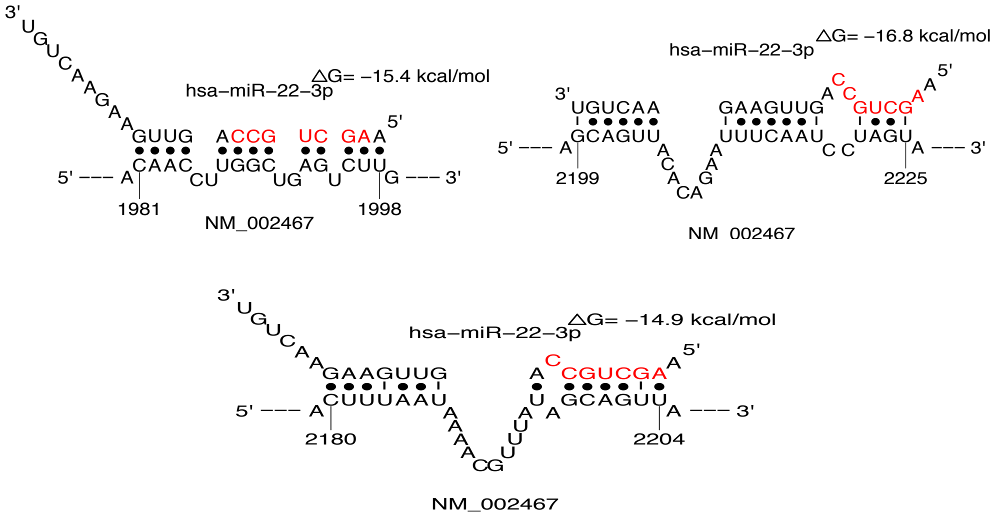


| miRNA | Site_Position | Hybrid Conformation | LogitProb† |
|---|---|---|---|
| hsa-miR-22-3p | 2313-2340 | View | 0.792 |
| hsa-miR-22-3p | 2180-2204 | View | 0.765 |
| hsa-miR-22-3p | 2199-2225 | View | 0.711 |
| hsa-miR-22-3p | 1981-1998 | View | 0.653 |

b

5'UTR

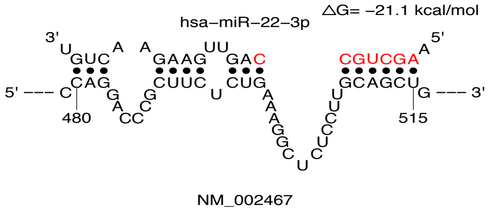


| miRNA | Site_Position | Hybrid Conformation | LogitProb† |
|---|---|---|---|
| hsa-miR-22-3p | 480-515 | View | 0.708 |

**Supplementary Figure S3: In silico prediction of miR-22 binding sites on MYC:** Predicted binding sites on 3'UTR (**a**) and 5'UTR **(b)** binding sites of MYC according to STarMir

SUPPLEMENTARY FIGURE S4

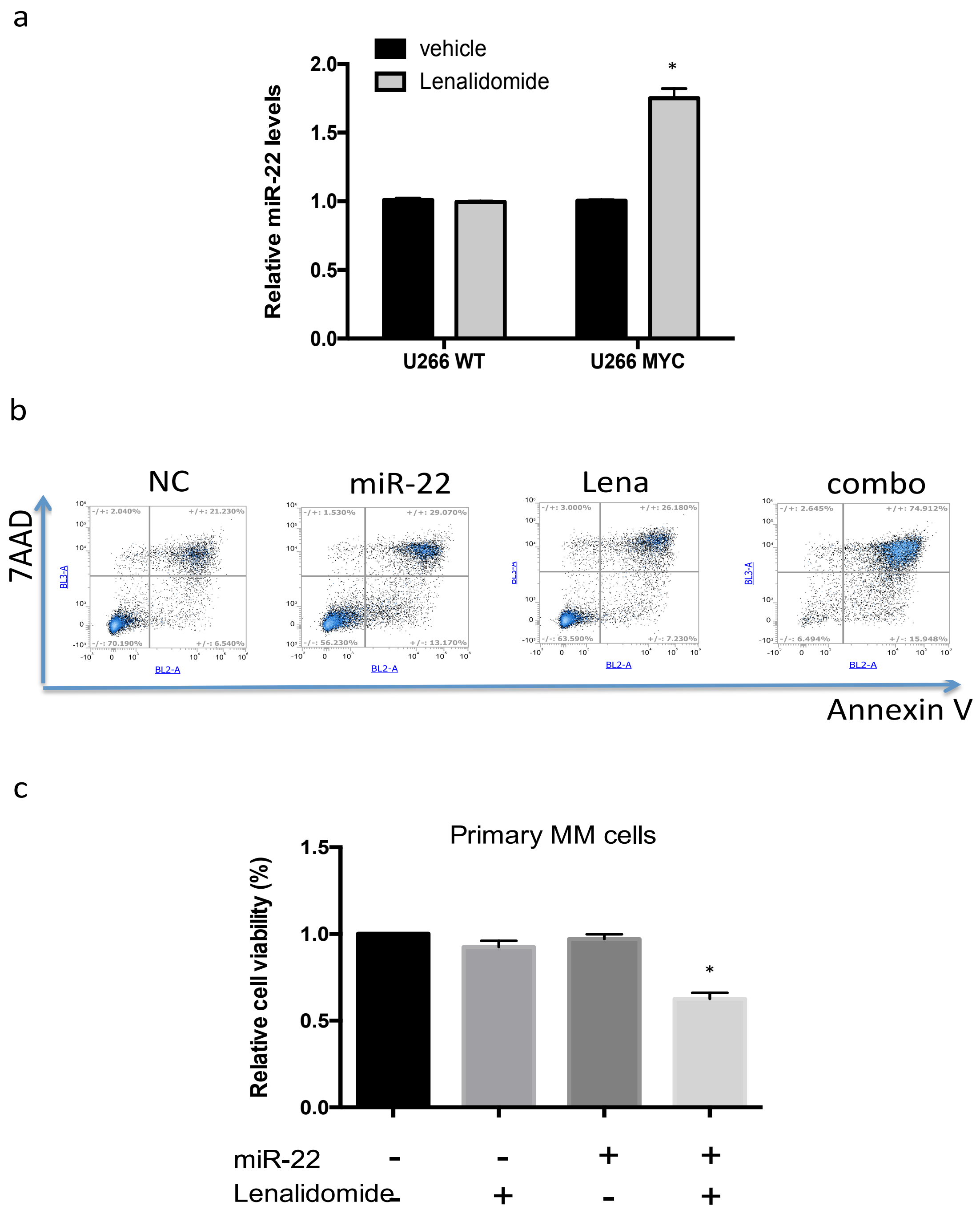


**Supplementary Figure S4: miR-22 sensitizes MM cell lines and primary cells to Lenalidomide:** a) U266 WT and U266 MYC overexpressing cells were treated with vehicle or Lenalidomide 10 µM for 72h and then miR-22 expression was evaluated by qRT-PCR. The results are shown as average miR-22 expression levels after normalization with RNU44 and ΔΔCt calculations. b) ABZB cells were transfected with miR-22 and after 24h, were treated with Lenalidomide 10 uM or vehicle.

Apoptosis was evaluated after 72h from Lenalidomide treatment. c) Primary relapsed MM cells n=3 Patients) were transfected with miR-22 and after 24h, were treated with Lenalidomide 10 uM or vehicle. Cell viability was evaluated after 48h from Lenalidomide treatment
Results are average ±SD of three independent experiments performed in triplicate.
*P<0.05

SUPPLEMENTARY FIGURE S5

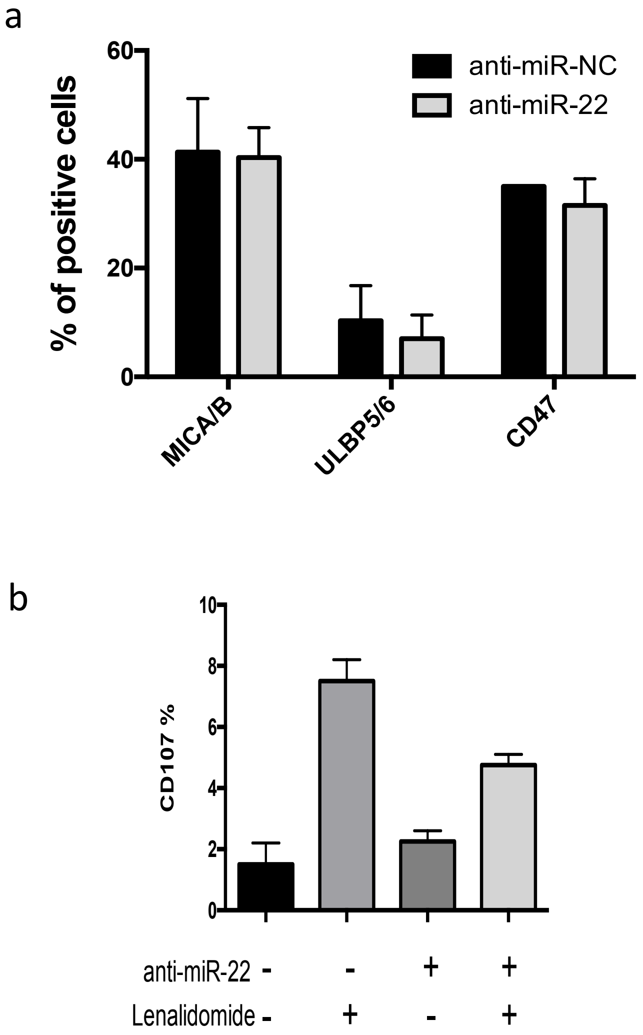


**Supplementary Figure S5: miR-22 inhibition antagonizes NK activation against MM cells: a)** flow cytometry of representative NK activating and inhibiting ligands performed on AMO1 cells transfected with miR-22 inhibitor or miR-NC. b) Percentage of CD107a positivity in effector cells (PBMCs) co-cultured with ABZB, after transfection with miR-22 inhibitor or miR-NC.

Results are average ±SD of three independent experiments performed in triplicate.
*$P<0.05$
